# A Review of Surface Water Quality Models

**DOI:** 10.1155/2013/231768

**Published:** 2013-06-17

**Authors:** Qinggai Wang, Shibei Li, Peng Jia, Changjun Qi, Feng Ding

**Affiliations:** Appraisal Center for Environment and Engineering, Ministry of Environmental Protection, Beijing 100012, China

## Abstract

Surface water quality models can be useful tools to simulate and predict the levels, distributions, and risks of chemical pollutants in a given water body. The modeling results from these models under different pollution scenarios are very important components of environmental impact assessment and can provide a basis and technique support for environmental management agencies to make right decisions. Whether the model results are right or not can impact the reasonability and scientificity of the authorized construct projects and the availability of pollution control measures. We reviewed the development of surface water quality models at three stages and analyzed the suitability, precisions, and methods among different models. Standardization of water quality models can help environmental management agencies guarantee the consistency in application of water quality models for regulatory purposes. We concluded the status of standardization of these models in developed countries and put forward available measures for the standardization of these surface water quality models, especially in developing countries.

## 1. Signature of Water Quality Models

Water quality models can be effective tools to simulate and predict pollutant transport in water environment [1–3], which can contribute to saving the cost of labors and materials for a large number of chemical experiments to some degree. Moreover, it is inaccessible for on-site experiments in some cases due to special environmental pollution issues. Therefore, water quality models become an important tool to identify water environmental pollution and the final fate and behaviors of pollutants in water environment [3]. These construction projects such as petrochemical, hydrological, and paper-making projects can bring serious effects on aquatic environment after enforcement [4, 5]. Therefore, these environmental effects have to be simulated, predicted, and assessed using numerical models before these construction projects are implemented. These modeling results under different pollution scenarios using water quality models are very important components of environmental impact assessment. Moreover, they are also the important basis for environmental management decisions as they not only provide data assistance for environmental management agencies to authorize the construction projects but also provide technical supports for water environmental protection agencies [6, 7]. Whether these model results are right or not can greatly impact the reasonability and scientific significance of the authorized construction projects and the availability of pollution control measures. 

With the development of model theory and the fast-updating computer technique [8], more and more water quality models have been developed with various model algorithms [3, 4]. Up to date, tens of types of water quality models including hundreds of model softwares have been developed for different topography, water bodies, and pollutants at different space and time scales [3, 9]. However, there are often big differences between these modeling results due to different theories and algorithms of these models, which can lead to the insistency of the predicted results using different models, and thus bringing different environment management decisions as these modeling results cannot be referred or compared to each other [10].

The uniform model standardization system has not been established yet in most developing countries [9, 11], which limits the wide applications of these models to environmental management due to no references and comparisons among different modeling results. Therefore, it is very necessary for most developing countries to better understand the availability and precisions of different water quality models and their methods of calculation and calibration and progress in the model standardization in order to apply effectively these models and form a good model regulation system [11, 12]. In particular, this work can contribute to making better environmental management policies and authorizing reasonable construction projects.

## 2. Development of Surface Water Quality Models

Surface water quality models have undergone a long period of development since Streeter and Phelps built the first water quality model (S-P model) to control river pollution in Ohio state of the US [13]. Surface water quality models have made a big progress from single factor of water quality to multifactors of water quality, from steady-state model to dynamic model, from point source model to the coupling model of point and nonpoint sources, and from zero-dimensional mode to one-dimensional, two-dimensional, and three-dimensional models [31, 32]. More than 100 surface water quality models have been developed up to now. Cao and Zhang [11] classified these models based on water body types, model-establishing methods, water quality coefficient, water quality components, model property, spatial dimension, and reaction kinetics. However, each surface water quality model has its own constraint conditions [33]. Therefore, water quality models still need to be further studied to overcome the shortcomings of these current models. Generally, the surface water quality models have undergone three important stages since 1925 to now.

### 2.1. The Primary Sage (1925–1965)

Water quality of water bodies has been paid much more attention to at this stage. The water quality models focused on the interactions among different components of water quality in river systems as affected by living and industrial point source pollution [9, 11, 34]. Like hydrodynamic transmission, sediment oxygen demand and algal photosynthesis and respiration were considered as external inputs, whereas the nonpoint source pollution was just taken into account as the background load [35, 36]. 

At the beginning of this stage (from 1925 to 1965), the simple BOD-DO bilinear system model was developed and achieved a success in water quality prediction, and the one-dimensional model was applied to solve pollution issues in rivers and estuaries [33]. After that, most researchers modified and further developed the Streeter-Phelps models (S-P models). For example, Thomas Jr. [14] believed that BOD could be reduced without oxygen consumption due to sediment deposition and flocculation, and the reduction rate was proportional to the number of remained BOD; thus, the flocculation coefficient was introduced in the steady-state S-P model to distinguish the two BOD removal pathways. O'Connor [15] divided BOD parameter into carbonized BOD and nitrified BOD and added the effects of dispersion based on the equation. Dobbins-Camp [16, 17] added two coefficients, including the changing rate of BOD caused by sediment release and surface runoff as well as the changing rate of DO controlled by algal photosynthesis and respiration, to Thomas's equation. 

### 2.2. The Improving Stage (1965–1995)

From 1965 to 1970, water quality models were classified as six linear systems and made a rapid progress based on further studies on multidimensional coefficient estimation of BOD-DO models. The one-dimensional model was updated to a two-dimensional one which was applied to water quality simulation of lakes and gulfs [37, 38]. Nonlinear system models were developed during the period from 1970 to 1975 [39]. These models included the N and P cycling system, phytoplankton and zooplankton system and focused on the relationships between biological growing rate and nutrients, sunlight and temperature, and phytoplankton and the growing rate of zooplankton [35, 37, 39]. The finite difference method and finite element method were applied to these water quality models due to the previous nonlinear relationships and they were simulated using one- or two-dimensional models. 

After 1975, the number of state variables in the models increased greatly, and the three-dimensional models were developed at this stage, and the hydrodynamic mode and the influences of sediments were introduced to water quality models [40, 41]. Meanwhile, water quality models were combined with watershed models to consider nonpoint source pollution input as a variable [42, 43]. The effects of sediments were coped with inner interaction processes of the models [43]; so, the sediment fluxes could vary accordingly under different input conditions. Therefore, the water quality management policies were greatly improved due to more constraint conditions and nonpoint source pollution simulation at watershed scale. The typical models including QUAL models [18, 19], MIKE11 model [22], and WASP models [23, 44] were developed and used at this stage. Meanwhile, the one-dimensional OTIS model developed by USGS was also applied to water quality simulation [45, 46]. 

### 2.3. The Deepening Stage (after 1995)

Nonpoint source pollution has been reduced due to strong control in developed countries. However, the dry and wet atmospheric deposition such as organic compounds, heavy metals, and nitrogen compounds showed increasing effects on water quality of rivers [47–49]. Although nutrients and toxic chemical materials depositing to water surface have been included in model framework, these materials not only deposited directly on water surface but also they can be deposited on the land surface of a watershed and sequentially transferred to water body [20, 50], which has been an important pollutant source. From the viewpoint of management demands, an air pollution model has to be developed to introduce this proceed in the model, indicating that the static or dynamic atmospheric deposition should be related to a given watershed [51]. Therefore, at this stage, some air pollution models were integrated to water quality models to evaluate directly the contribution of atmospheric pollutant deposition [20]. 

With the exception of the typical models such as QUAL 2K model [52], WASP 6 model [24], QUASAR model [25, 53], SWAT model [21], and MIKE 21 [26] and MIKE 31 models [27] (Table 1), other water quality models have also been developed to simulate complicated water environmental conditions. For example, Whitehead et al. [54] developed a semidistributed integrated nitrogen model (INCA) based on the effects of atmospheric and soil N inputs, land uses, and hydrology. More recently, Fan et al. [55] integrated QUAL 2K water quality model and HEC-RAS model to simulate the impact of tidal effects on water quality simulation. For the integration of point and nonpoint sources, the US Environmental Protection Agency (USEPA) developed a multipurpose environmental analysis system (BASINS), which makes it possible to assess quickly large amounts of point and nonpoint source [28]. Meanwhile, the USEPA also listed the EFDC model as a tool for water quality management.

Among the previously mentioned surface water quality models, these models including the Streeter-Phelps model, QUASAR model, QUAL model, WASP model, CE-QUAL-W 2 model, BASINS model, MIKE model, and EFDC model were widely applied worldwide [56, 57]. Recently, Kannel et al. [58] concluded that these public domain models (e.g., QUAL2EU, WASP7, and QUASAR) are the most suitable for simulating dissolved oxygen along rivers and streams. Generally, most developed countries (especially the US or European countries) have developed better and advanced surface water quality models [22, 27, 28, 30]. Some surface water quality models have also been established in some universities or institutes of China over the past years [11], but these models were still not widely utilized like MIKE models, EFDC model, and WASP models [59, 60].

## 3. Standardization of Surface Water Quality Models

Water quality models should be more available, standardized, and reliable when they are utilized to aid the important and valid reports (e.g., environmental impact assessment report). Therefore, it is very necessary for environmental management agencies to mandate or list some water quality models in order to guarantee the consistency of water quality models for regulatory purposes [61]. The models can be regulated and standardized through these pathways such as the establishment of the national model assessment indicator and validating system, published articles, workshops, or setting up local workgroup [62]. For example, The USEPA holds regular academic conferences on water quality models to identify and update regulatory models [62]. The European Union organizes regular workshops on the consistency of water quality models to evaluate the regulatory models. Moreover, the standardized models should be able to be downloaded free and have open origin codes. 

Special research institutes of water quality models have been built to do a lot of researches on the regulation and standardization of water quality models in some regions or countries [62, 63]. They recommended some prediction models based on the requirements of environmental management. Compared to other countries, most water environmental models have been standardized in the US. The Water Science Center belonging to the USEPA focuses on the following studies regarding water resources management and conservation, the theory and methods applied in water environments, numerical models, calculating tools, and databanks. Meanwhile, the USEPA also provides foundations for some universities, institutes, or companies to develop and compare related models and finish a series of research reports. In 2002, the USEPA mandated the Guidance for Quality Assurance Project Plans for Models, and some advices and guidance principles were given for the applications of water quality models in this guidance [64]. Additionally, the USEPA also authorized Tetra Tech Inc. to do the project of TMDL Model Evaluation and Research Needs, through which the modeling capacity, availability, and scopes of more than 60 models have been evaluated and compared using detailed appraisal forms [65]. Based on the above researches, the USEPA finally published the Guidance on the Development, Evaluation, and Application of Environmental Models in 2009. This guidance introduces concisely the characteristics and appropriate environment process modeling of these surface water environment models such as HSPF model, WASP model, and QUAL2E model and also gave the website links for more details of these models. The best practices for model evaluation are also appended to this guidance, which describes the methods, objectives, and procedures of model evaluation in detail [66]. Besides the guidance, the Council for Regulatory Environmental Modeling of the USEPA provides the model banks on its website. The United States Geological Survey, Federal Emergency Management Agency, and the United States Army Corps of Engineers also have similar model banks and detailed introduction for different types of models. The USEPA recommended its own developed models and those models developed by other research institutes or companies, but an announcement has been provided in the recommendation report that the recommended models do not denote that they have been authenticated by the USEPA [66]. The USEPA only suggested how to select appropriate models under different environmental conditions as each model has its own appropriate scope and scale. However, Kannel et al. [58] pointed out that the choice of a model depends upon availability of time, financial cost, and a specific application.

Similarly special research institute of model development and evaluation has been set up by the United Kingdom Environment Agency (UKEA). This institute helped the UKEA finish the Framework for Assessing the Impact of Contaminated Land on Groundwater and Surface Waterand and put forward the procedure, method, and prediction models of surface water environmental impact assessment of potential pollution sources, which can assess the influencing degree of pollution sources on water environment. The Her Majesty's Inspectorate of Pollution (HMIP) recommended 54 surface water quality models and limiting conditions for rivers, lakes, reservoirs, estuaries, and sea pollution assessment. Aspinwall and Company Limited recommended 11 models for different conditions including 1 one-dimensional model, 4 two-dimensional models, and 6 three-dimensional models, of which 11 models for steady-state simulation and 10 models for dynamic simulation [67]. In Korea, the Ministry of Environment made a general plan for water environmental management in 2006, which described 6 water quality prediction models in detail and recommended a series of numerical models including widely-used Qual2E model and EFDC model [68]. The MIKE models and Tuflow model were widely applied to predict surface water quality in Australia. MIKE models were adapted in Denmark to solve some issues in these fields such as ecology, environmental chemistry, water resources, hydraulic engineering, and hydrological dynamics. In China, the Delft 3D hydrological dynamic-water quality model has been used to simulate water environmental quality in Hong kong since 1970s and now become the standard model of Hong kong Environment Agency. Taiwan Environmental Protection Bureau issued the guidance on methods of water quality assessment of rivers and environmental impact assessment and provided a water quality model list for different conditions in this guidance. The Ministry of Environmental Protection of China formally published the Technical Guidelines for Environmental Impact Assessment (Surface water Environment) in 1993 and recommended some numerical models for rivers, lakes, estuaries, and marine environment under different conditions [69]. However, the standardized numerical models in China are still not provided yet up to date. Most models such as MIKE models, EFDC model, and Delft 3D model have been applied to simulate water environmental quality in most institutes of environmental impact assessment [70, 71]. However, little information is available on the differences in model results from different models and the suitability and parameter sensitivity of these models. Moreover, it is also an urgent task to standardize some numerical models to compare the modeling results among different regions efficiently. Additionally, Moriasi et al. [72] suggested to develop the consistent framework of model calibration and validation guidelines, as it is difficult to compare modeling results from different studies with different calibration and validation methods.

## 4. Measurements for the Standardization of Surface Water Quality Models

The appraisal techniques of the standardization of water quality models and their authentication system can provide an important scientific basis for the development of software informatization for water quality models and environmental impact assessment [68]. To improve the standardization of surface water quality models, the best way is to understand fully the status, progress, frame structure, assessing indicators, and authentication system of the standardization system of surface water quality models in developed countries, especially in some European or North American countries. Based on the previously mentioned, it is necessary for environmental management agencies of those countries without standardization models of water environmental quality to develop their own construction and frame structure of standardized model system of surface water quality, screen assessing indicators, procedures, and methods to establish their own authentication and standardization system for surface water quality models.

**Figure 1:**
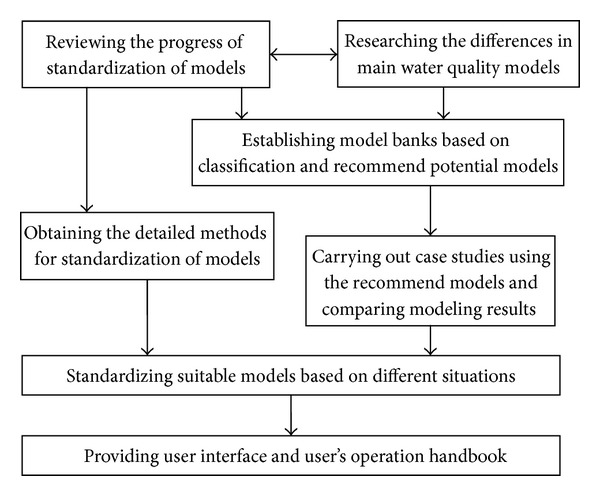
Flow chart of the standardization of water quality models.

The specific measures for the standardization of surface water quality models are given as follows (Figure 1).

1. To research the water quality models which are widely used in the fields of surface water environmental impact assessment to know well the model mechanisms, suitable conditions, appropriate scopes, model parameters, stability, and the differences in modeling results.
2. To develop case bank and data bank for surface water quality models through indoor experiments, case collection, and field monitoring.
3. To compare the modeling results among different models and to conclude and analyze the input and output files, equations, theories, frames, and calculating methods of water quality models based on some case studies.
4. To provide the screening indicators and appraisal methods for water quality models to establish the appraisal authentication system of these models and standardize the standard interfaces of input and output data for these models. To standardize some water quality models and list the standardized models for environmental impact assessment based on each country's actual conditions.
5. To give the parameter calibration and validation methods and the access, sources, and recommended values of these parameters and put forward some standard proposals for typical model parameters considering the actual conditions of each country.
6. To provide user interface of model graphs in native language and publish detailed model operation handbook including model inputs (data access, data processing), model structure, model calibration, model validation, parameter assessment, and model outputs.

## 5. Conclusions

Water quality models are very important to predict the changes in surface water quality for environmental management in the world. Worldwide, hundreds of surface water quality models have been developed. Moreover, some developed countries have mandated the guidance on water environmental quality assessment and provided some regulated models for surface water quality simulation. Therefore, it is very necessary for most developing countries to standardize some widely used water quality models for efficient environmental impact assessment. However, it is also a big challenge to standardize these models based on their own countries' actual conditions as a lot of investigations and researchers are still needed.

## Figures and Tables

**Table 1:** Main surface water quality models and their versions and characteristics.

Models	Model version	Characteristics
Streeter-Phelps models	S-P model [13]; Thomas BOD-DO model [14]; O'Connor BOD-DO model [15]; Dobbins-Camp BOD-DO model [16, 17]	Streeter and Phelps established the first S-P model in 1925. S-P models focus on oxygen balance and one-order decay of BOD and they are one-dimensional steady-state models.

QUAL models	QUAL I [11]; QUAL II [18]; QUAL2E [19]; QUAL2E UNCAS [19]; QUAL 2K [20, 21]	The USEPA developed QUAL I in 1970. QUAL models are suitable for dendritic river and non-point source pollution, including one-dimensional steady-state or dynamic models.

WASP models	WASP1-7 models [22, 23]	The USEPA developed WASP model in 1983. WASP models are suitable for water quality simulation in rivers, lakes, estuaries, coastal wetlands, and reservoirs, including one-, two-, or three-dimensional models.

QUASAR model	QUASAR model [11, 24, 25]	Whitehead established this model in 1997. QUASAR model is suitable for dissolved oxygen simulation in larger rivers, and it is a one-dimensional dynamic model including PC_QUA SAR, HERMES, and QUESTOR modes.

MIKE models	MIKE11 [22]; MIKE 21 [26]; MIKE 31 [27]	Denmark Hydrology Institute developed these MIKE models, which are suitable for water quality simulation in rivers, estuaries, and tidal wetlands, including one-, two-, or three dimensional models.

BASINS models	BASINS 1 [11, 28]; BASINS 2 [11, 28]; BASINS 3 [11, 28]; BASINS 4 [28]	The USEPA developed these models in 1996. BASINS models are multipurpose environmental analysis systems, and they integrate point and nonpoint source pollution. BASINS models are suitable for water quality analysis at watershed scale.

EFDC model	EFDC model [29, 30]	Virginia Institute of Marine Science developed this model. The USEPA has listed the EFDC model as a tool for water quality management in 1997. EFDC model is suitable for water quality simulation in rivers, lakes, reservoirs, estuaries, and wetlands, including one-, two-, or three-dimensional models.
